# Publisher Correction: Mapping microscale wetting variations on biological and synthetic water-repellent surfaces

**DOI:** 10.1038/s41467-018-03042-0

**Published:** 2018-02-09

**Authors:** Ville Liimatainen, Maja Vuckovac, Ville Jokinen, Veikko Sariola, Matti J. Hokkanen, Quan Zhou, Robin H. A. Ras

**Affiliations:** 1 Department of Electrical Engineering and Automation, Aalto University School of Electrical Engineering, Maarintie 8, 02150 Espoo, Finland; 2 Department of Applied Physics, Aalto University School of Science, Puumiehenkuja 2, 02150 Espoo, Finland; 3 Department of Chemistry and Materials Science, Aalto University School of Chemical Technology, Tietotie 3, 02150 Espoo, Finland; 4 Faculty of Biomedical Sciences and Engineering, Tampere University of Technology, Korkeakoulunkatu 3, 33720 Tampere, Finland; 5 Department of Bioproducts and Biosystems, Aalto University School of Chemical Engineering, Kemistintie 1, 02150 Espoo, Finland

Correction to: *Nature Communications* 10.1038/s41467-017-01510-7, published online 27 November 2017

The original version of this Article contained an error in Fig. 2b. The *y*-axis label in Fig. 2b was incorrectly labelled ‘Snap-in force (μN)’. In the correct version, the axis is labelled ‘Snap-in force (nN)’.

In the original version of this Article, the “Butterfly wings” section of the Methods incorrectly stated the name of the butterfly as ‘Golden bird’. In the correct version, the name of the butterfly is ‘Golden birdwing’.

These have now been corrected in both the PDF and HTML versions of the article.

