# Supplementary material for: Mapping microscale wetting variations on biological and synthetic water-repellent surfaces
Source: Nat Commun. 2017 Nov 27;8:1798. doi: 10.1038/s41467-017-01510-7 (PMC5702616; doi:10.1038/s41467-017-01510-7)
Supplement: Supplementary file 1 — Supplementary Information [file 41467_2017_1510_MOESM1_ESM.pdf]

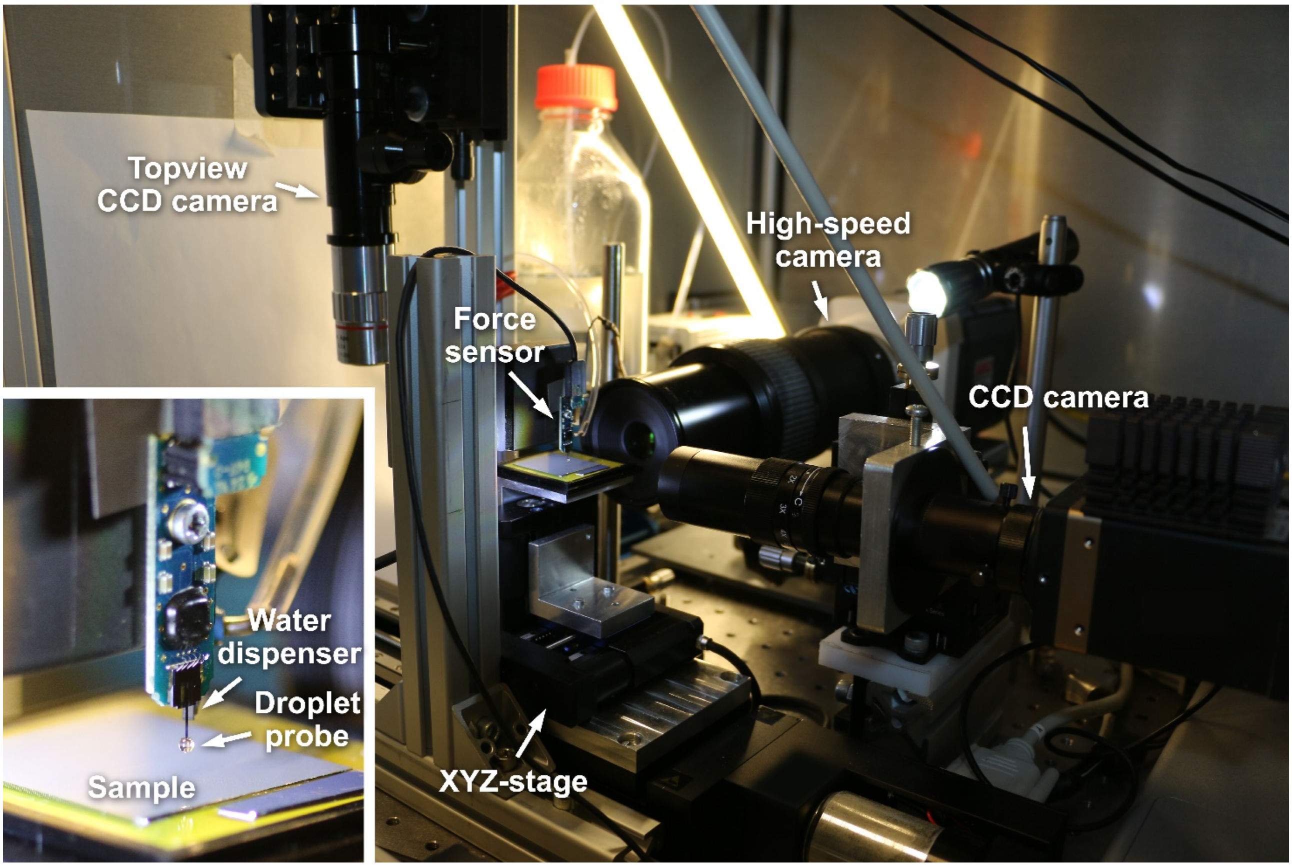

Supplementary Figure 1. Droplet adhesion force microscopy setup. Inset shows a close-up of the droplet probe at the end of the force sensor.

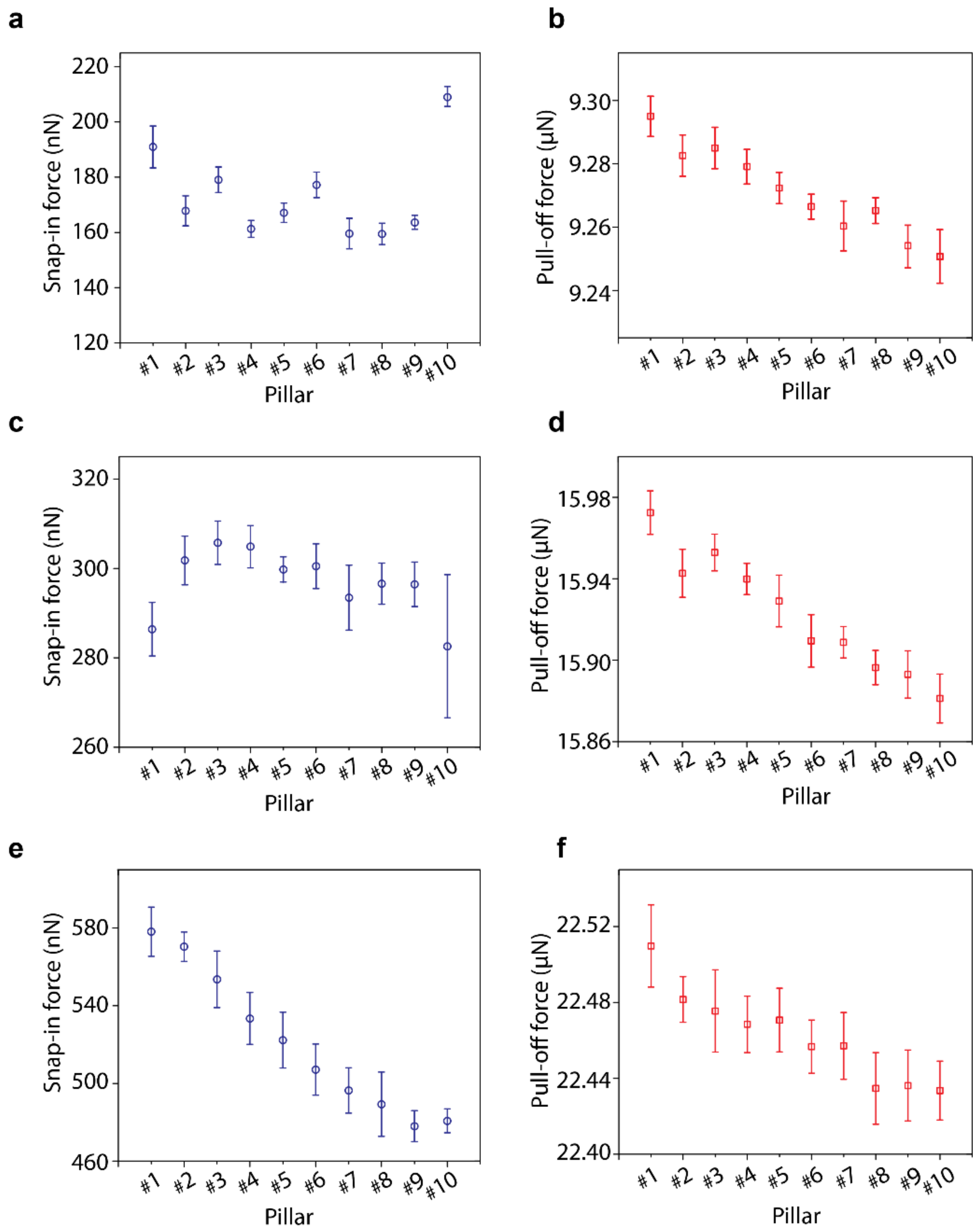

Supplementary Figure 2. Accuracy and repeatability of droplet adhesion forces. Snap-in and pull-off forces, respectively, on **a,b** 20 μm radius pillars, **c,d** 35 μm radius pillars and **d,e** 50 μm radius pillars. Error bars present standard deviation for ten repetitions on each pillar.

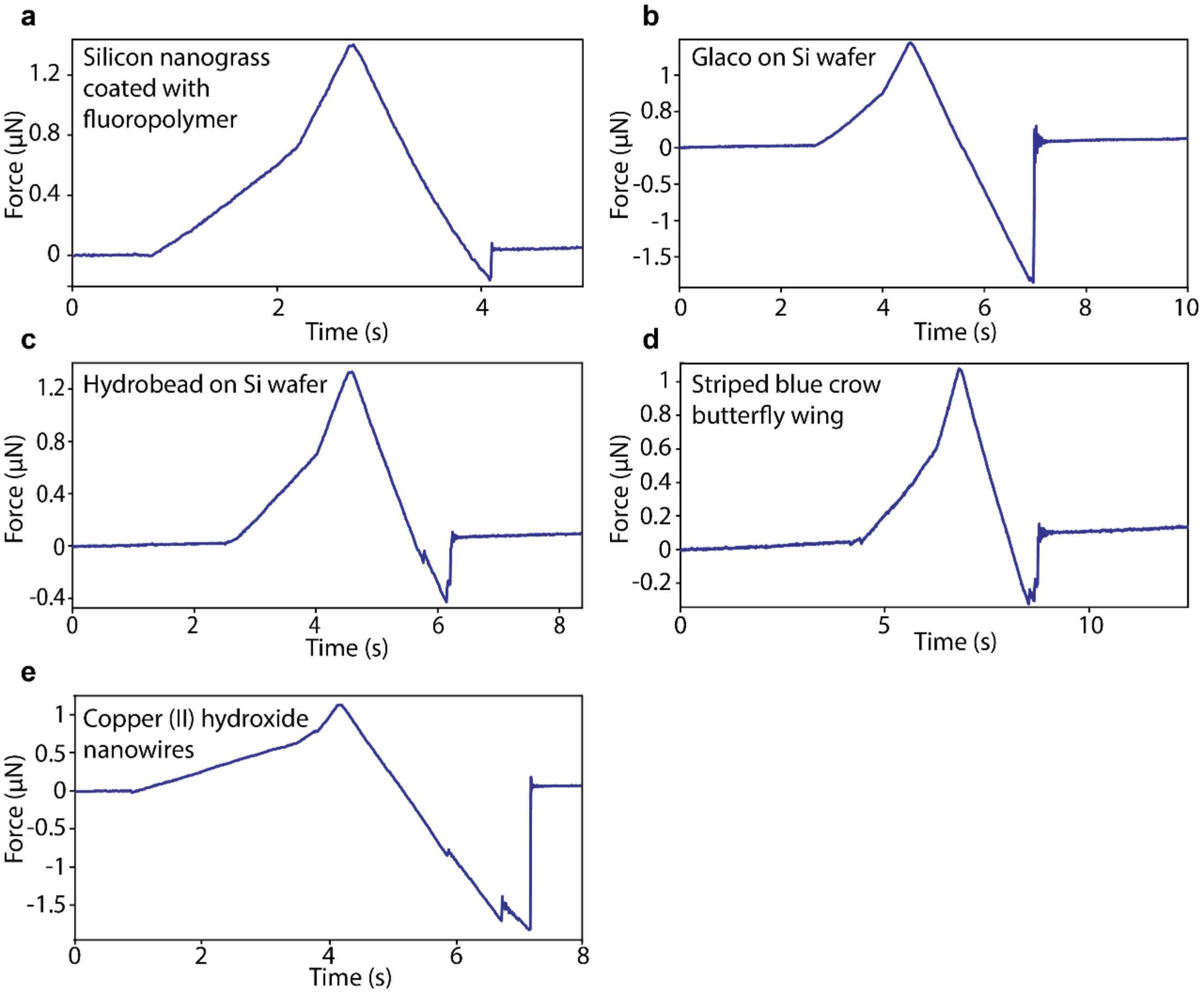

Supplementary Figure 3. Force curves for **a,** Silicon nanograss coated with fluoropolymer, **b,** Glaco on Si wafer, **c,** Hydrobead on Si wafer, **d,** striped blue crow butterfly wing and **e,** copper (II) hydroxide nanowires.

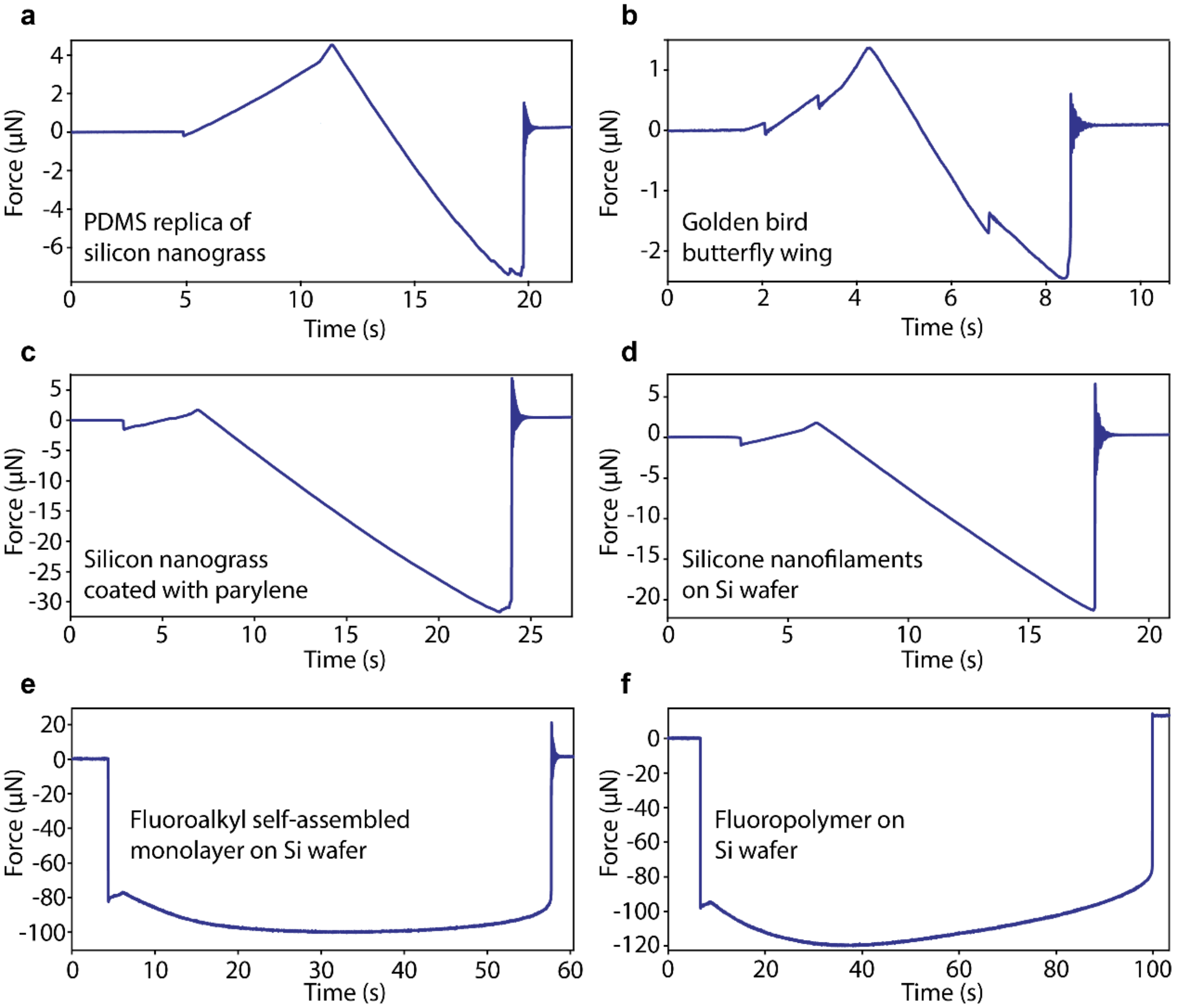

Supplementary Figure 4. Force curves for **a,** PDMS replica of silicon nanograss, **b,** golden bird butterfly wing, **c,** silicon nanograss coated with parylene, **d,** silicone nanofilaments on Si wafer, **e,** fluoroalkyl self-assembled monolayer on Si wafer and **f,** fluoropolymer on Si wafer. Note the volume loss visible in panel **f**, where the final force level is significantly higher than the initial value. The flat surface has a macroscopically measured receding contact angle of less than 90° (see Supplementary Table 2) and leaves a droplet behind on the surface at pull-off.

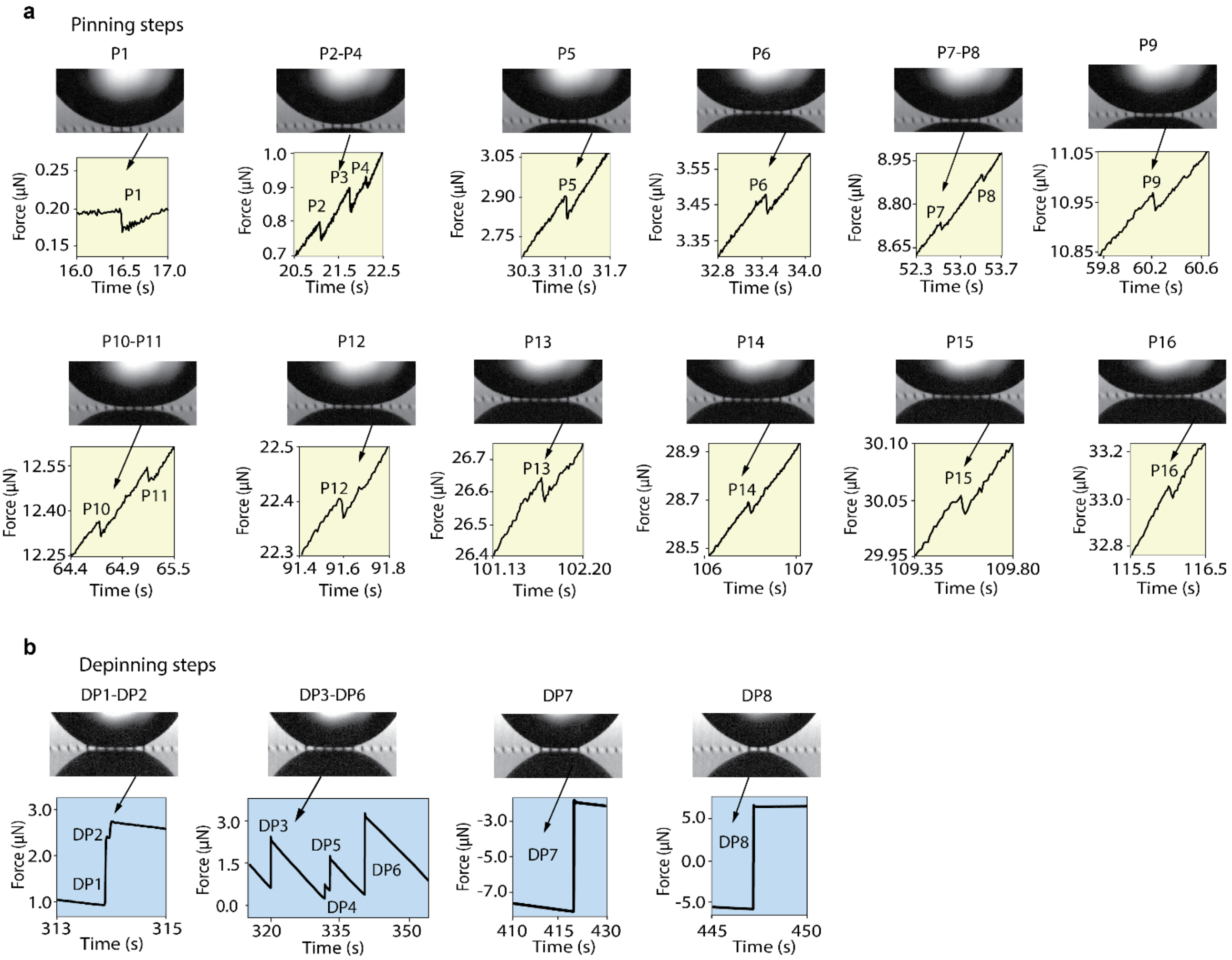

Supplementary Figure 5. Multiple wetting steps on a pillar array surface. a, All 16 consecutive pinning steps while droplet advances (sample surface moving up) and **b,** 8 depinning steps while droplet recedes on the surface (droplet evaporating, sample surface not moving). Side views of droplet contact area correspond to the pinning/depinning step pointed by arrow.

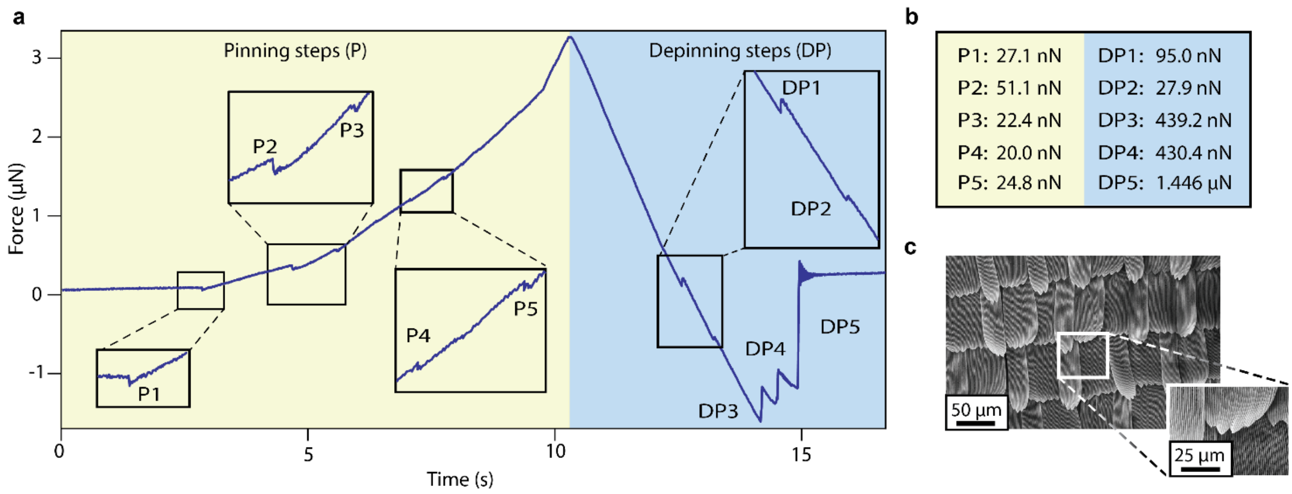

Supplementary Figure 6. Multiple wetting steps on striped blue crow butterfly wing. a, Droplet adhesion force curve with insets which show multiple pinning (P1-P5) steps while droplet advances (sample surface is moving up) and depinning (DP1-DP5) steps while droplet recedes (sample surface is moving down) with **b,** corresponding force values of individual pinning/depinning steps. **c,** SEM image of butterfly wing.

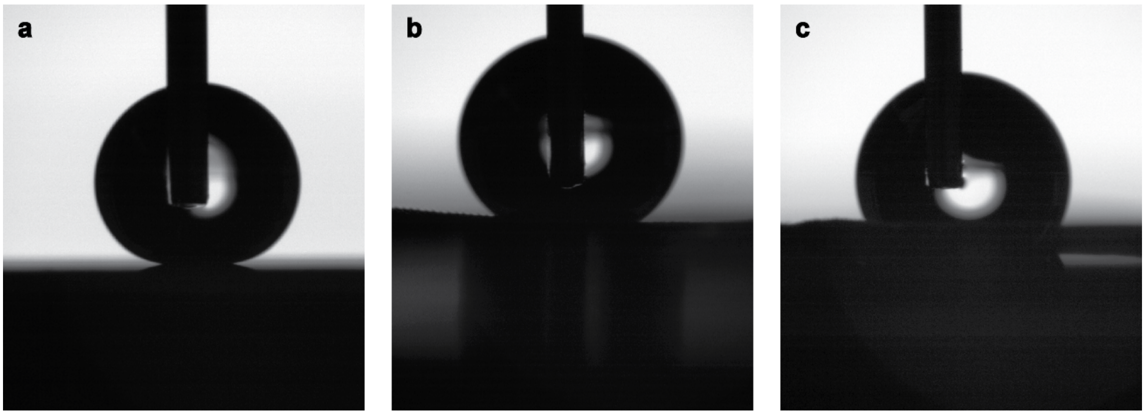

Supplementary Figure 7. Comparison of contact angle measurements **a,** on a flat surface, where surface baseline is clearly visible, and **b-c**, on non-flat biological surfaces where the surface baseline is obscured. **a,** Silicon nanograss coated with fluoropolymer; **b,** striped blue crow butterfly wing and **c,** golden bird butterfly wing.

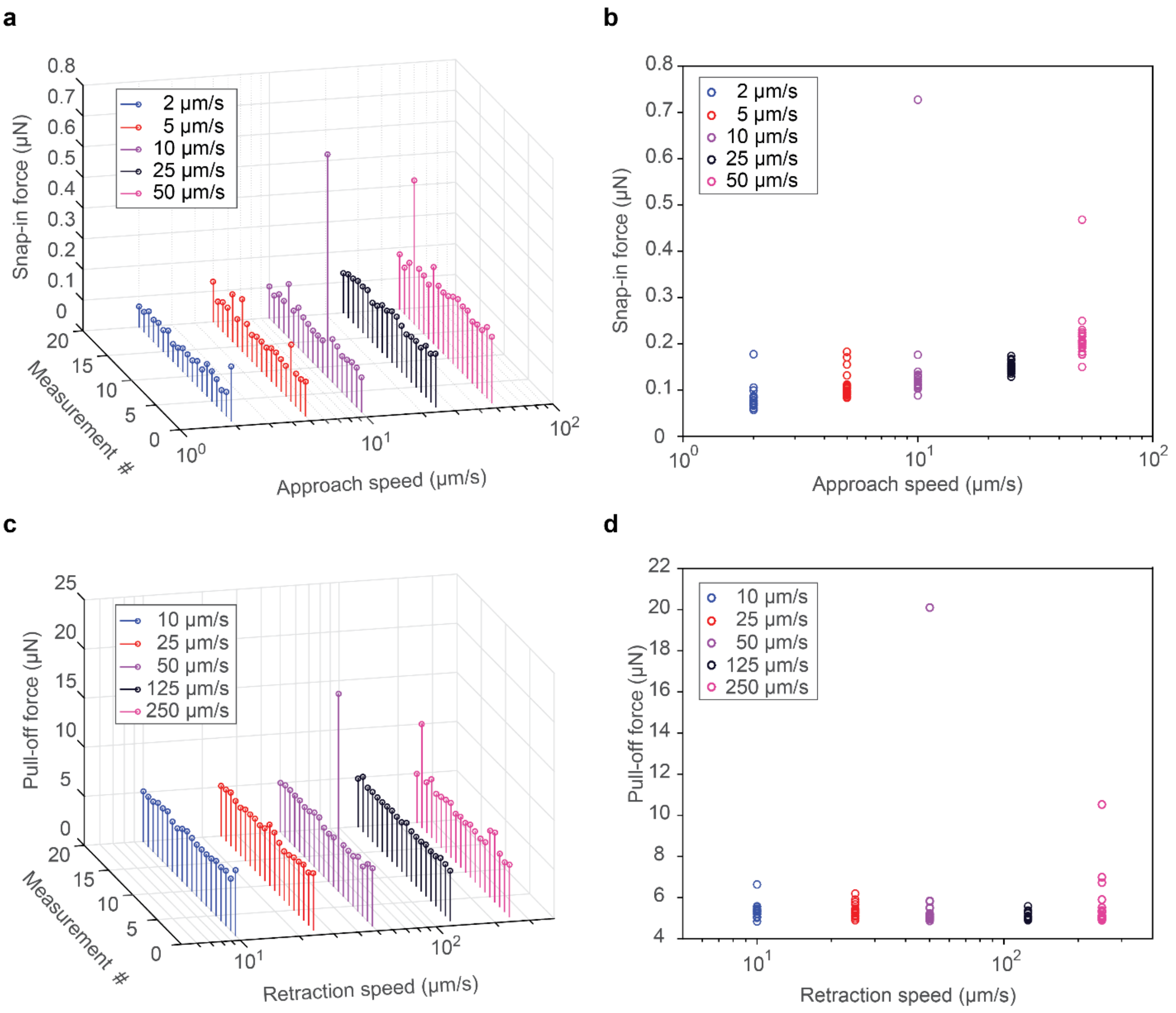

Supplementary Figure 8. Droplet adhesion forces vs. different stage movement speeds. **a,b** Snap-in force vs. approach speed and **c,d** pull-off force vs. retraction speed. The surface is a fluoropolymer-coated silicon nanograss (slightly less superhydrophobic than the one listed in Supplementary Table 2). The measurements were carried out on 100 different locations on the sample. Approach and retraction speed are shown to have little or no effect on measured force.

Supplementary Table 1. Analysis of variance (ANOVA) of the force on silicon micropillars with different pillar radii. Notice the pooled standard deviation of all measurements is in the same order of magnitude for all pillar radii, and is comparable to the sensor noise (5 nN).

	10 μm	20 μm	35 μm	50 μm
Snap-in force (mean ± pooled std)	125.9 ± 6.6 nN	173.5 ± 4.9 nN	296.8 ± 7.4 nN	520.9 ± 12.9 nN
F-value (ANOVA)	5.72	108	10.3	81.4
p-value (ANOVA)	3.01×10^{-6}	2.92×10^{-44}	9.32×10^{-11}	2.36×10^{-39}
Pull-off force (mean ± pooled std)	4.6064 μN ± 5.3 nN	9.2710 μN ± 6.6 nN	15.9226 μN ± 11.2 nN	22.4624 μN ± 18.4 nN
F-value (ANOVA)	9.14	47.12	69.0	17.2
p-value (ANOVA)	1.05×10^{-9}	2.84×10^{-30}	1.55×10^{-36}	3.42×10^{-16}

Supplementary Table 2. Advancing contact angles, snap-in forces, receding contact angles and pull-off forces for various surfaces. Snap-in and pull-off forces are average values of measurements on 5 different spots with standard deviations, while advancing and receding contact angles are average values of measurements on 10 different spots with standard deviations.

Sample	Advancing contact angle (°)	Snap-in force (nN)	Receding contact angle (°)	Pull-off force (nN)
Silicon nanograss coated with fluoropolymer	174.6 ± 4.5	-[a]	169.0 ± 7.0	218.5 ± 9.7
Glaco on Si wafer	169.7 ± 2.8	-[a]	163.0 ± 3.9	1526.0 ± 297.6
Hydrobead on Si wafer	169.3 ± 3.7	7.8 ± 1.9 [b]	168.2 ± 3.6	524.7 ± 195.5
Striped blue crow butterfly wing	-[c]	10.0 ± 5.3	-[c]	414.9 ± 188.9
Copper (II) hydroxide nanowires	158.5 ± 3.1	16.5 ± 5.4	156.0 ± 3.2	1626.8 ± 543.5
PDMS replica of silicon nanograss	157.8 ± 3.9	30.1 ± 16.2	152.8 ± 3.0	7515.0 ± 1114.4
Golden bird butterfly wing	-[c]	61.2 ± 58.0	-[c]	1329.8 ± 535.3
Silicon nanograss coated with parylene	151.2 ± 4.6	898.7 ± 185.2	142.6 ± 7.2	26005.5 ± 1089.2
Silicone nanofilaments on Si wafer	149.8 ± 5.0	927.0 ± 416.8	143.5 ± 5.2	19602.3 ± 3490.0
Fluoroalkyl self-assembled monolayer on Si wafer	123.2 ± 0.5	80963.8 ± 601.1	101.8 ± 0.5	103703.0.2 ± 3651.0
Fluoropolymer on Si wafer	110.9 ± 1.1	98739.3 ± 619.4	85.1 ± 0.4	135101.5 ± 438.1

[a] Snap-in force is smaller than sensor noise (Supplementary Figure 3).
[b] Mean of three measurements, two spots had snap-in force smaller than sensor noise.
[c] The butterfly wings are curved and obscure the surface baseline, making it not feasible to measure advancing and receding contact angles (Supplementary Figure 7).

Supplementary Note 1. Computational model of the snap-in and pull-off forces.

To compute the adhesive force resulting from the droplet between the probe disk and the substrate, we follow the general approach of H.-J. Butt et al. [1]. The axially symmetric geometry of the problem is shown in Supplementary Figure 9. We assume that the probe-to-substrate distance is changing slowly so that the droplet interface is always at equilibrium and that gravity can be neglected.

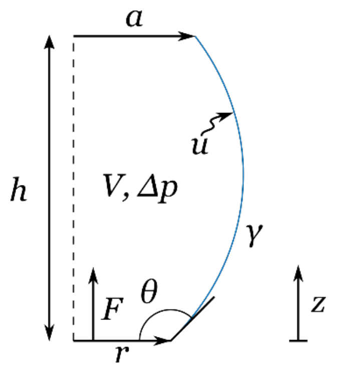

Supplementary Figure 9. Schematic of the problem. u: radial distance of the interface from the axis of symmetry as a function of z. a: radius of the probe disk, $a = 0.5$ mm. r: contact radius. θ: contact angle. h: height of the probe-substrate gap. F: force between the droplet and the substrate. γ: surface tension, $\gamma \approx 72$ mN m^{-1}. V: volume of the droplet. Δp: Laplace pressure.

In equilibrium, the droplet interface has a constant mean curvature and fulfills the Young-Laplace equation, given in cylindrical coordinates as

$$\frac{u''}{(1+u'^2)^{3/2}} - \frac{1}{u\sqrt{1+u'^2}} = -\Delta p/\gamma \qquad (1)$$

where $u = u(z)$ and u', u'' are the first and second derivatives with respect to z. Knowledge of the droplet shape allows computing F as a sum of capillary and Laplace pressure terms

$$F = 2\pi\gamma r sin\theta - \pi r^2 \Delta p \qquad (2)$$

A priori, we do not know the pressure Δp inside the droplet, but we know the volume V (constant):

$$V = \int_0^h \pi u^2 dz = W\rho^{-1}g^{-1} = \text{constant} \qquad (3)$$

where W is the weight of the droplet, $W = 15$ µN in our experiments, $\rho \approx 1000$ kg m^{-3} is the density of the liquid, and $g \approx 9.81$ m s^{-2} is the gravitational acceleration.

We consider two different, but closely similar, problems: 1) **Fixed radius problem**: Contact line on the substrate is pinned e.g. to the edge of a micropillar so that contact radius r is fixed. Contact angle θ may vary. 2) **Fixed angle problem**: Contact angle θ is fixed, e.g. on a perfectly flat substrate. Contact line is free to move i.e. r may vary.

We focus on solving the fixed radius problem; it is trivial to adapt the method to the fixed angle problem.

Problem statement

The fixed radius problem is a boundary value problem:

$$\begin{cases} \frac{u''}{(1+u'^2)^{3/2}} - \frac{1}{u\sqrt{1+u'^2}} = -\Delta p/\gamma, \ z \in (0,h) \\ u(0) = r \\ u(h) = a \\ \int_0^h \pi u^2 dz = V \end{cases} \quad (4)$$

where Δp is a free parameter. After solving Supplementary Equation 4, the force can be computed using (2) and the relation

$$\sin\theta = \frac{1}{\sqrt{1+u'^2(0)}} \quad (5)$$

Thus, the force F is a function of five variables

$$F = F(\gamma, h, r, a, V) \quad (6)$$

Non-dimensionalization

Note that Supplementary Equation 6 can be non-dimensionalized by rescaling everything by the powers of a and γ i.e.

$$\begin{aligned} \tilde{F} &= F\gamma^{-1}a^{-1} \\ \tilde{r} &= ra^{-1} \\ \tilde{V} &= Va^{-3} \\ \Delta\tilde{p} &= \Delta p a \gamma^{-1} \\ &\text{etc.} \end{aligned} \quad (7)$$

The ~ accent indicates non-dimensionalized values. This yields the non-dimensionalized problem

$$\begin{cases} \frac{\tilde{u}''}{(1+\tilde{u}'^2)^{3/2}} - \frac{1}{\tilde{u}\sqrt{1+\tilde{u}'^2}} = -\Delta\tilde{p}, \ \tilde{z} \in (0,\tilde{h}) \\ \tilde{u}(0) = \tilde{r} \\ \tilde{u}(\tilde{h}) = 1 \\ \int_0^{\tilde{h}} \pi \tilde{u}^2 d\tilde{z} = \tilde{V} \end{cases} \quad (8)$$

$$\tilde{F} = \tilde{F}(\tilde{h}, \tilde{r}, \tilde{V}) = \frac{2\pi\tilde{r}}{\sqrt{1+\tilde{u}'^2(0)}} - \pi\tilde{r}^2\Delta\tilde{p} \quad (9)$$

where we have used Supplementary Equations 2-7.

Shooting method

There are several ways to solve this boundary value problem; here we solve it using a shooting method, which is simple to implement, but numerically not very efficient. Instead of solving the boundary value problem, we consider the initial value problem

$$\begin{cases} \frac{\tilde{u}''}{(1+\tilde{u}'^2)^{3/2}} - \frac{1}{\tilde{u}\sqrt{1+\tilde{u}'^2}} = -\Delta\tilde{p}, \ \tilde{z} \in (0,\tilde{h}) \\ \tilde{u}(0) = \tilde{r} \\ \tilde{u}'(0) = x \end{cases} \quad (10)$$

We let

$$\mathbf{m}(x, \Delta\tilde{p}) = \begin{bmatrix} 1 - \tilde{u}(\tilde{h}; x, \Delta\tilde{p}) \\ \tilde{V} - \int_0^{\tilde{h}} \pi\tilde{u}^2(\tilde{z}; x, \Delta\tilde{p}) d\tilde{z} \end{bmatrix} \tag{11}$$

where $\tilde{u}(\tilde{z}; x, \Delta\tilde{p})$ is the solution to Supplementary Equation 10 using the values x and $\Delta\tilde{p}$. Solution to the system of equations

$$\mathbf{m} = \mathbf{0} \tag{12}$$

is the solution to the boundary value problem.

Simulink extension of Matlab is a graphical tool for the numerical integration of initial value problems such as Supplementary Equation 10. Simulink requires solving the equation for u''

$$\tilde{u}'' = (1 + \tilde{u}'^2)^{3/2} \left(\frac{1}{\tilde{u}\sqrt{1+\tilde{u}'^2}} - \Delta\tilde{p} \right) \tag{13}$$

The Simulink model of Supplementary Equation 13 is shown in Supplementary Figure 10. Conditionals stops were added to prevent the unphysical case $\tilde{u} < 0$ and singularities. We also compute the volume $V = \int \pi\tilde{u}^2 d\tilde{z}$ in the same model.

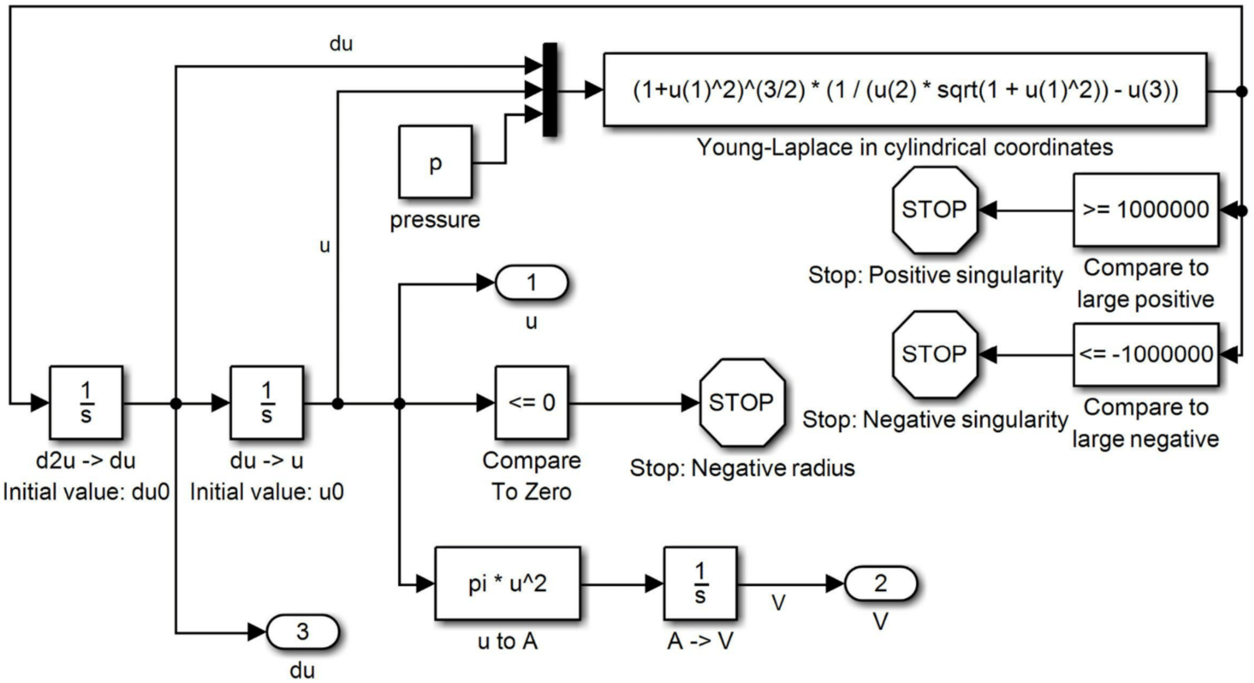

Supplementary Figure 10. Simulink model of the initial value Young-Laplace equation.

The code to solve Supplementary Equation 12 and to compute the force using Supplementary Equation 9 is given in Supplementary Software 1.

Snap-in forces on micropillars

It remains to determine $\tilde{h}$, which is different in the snap-in and pull-off cases, denoted respectively as $\tilde{h}_{\text{snap-in}}$ and $\tilde{h}_{\text{pull-off}}$. We first discuss the snap-in case. Our first assumption follows the approach in ref. [1]: we assume that just before snap-in, the droplet is a perfect spherical cap. At snap-in, the tip just touches the surface (Supplementary Figure 11) and wets the top of the micropillar.

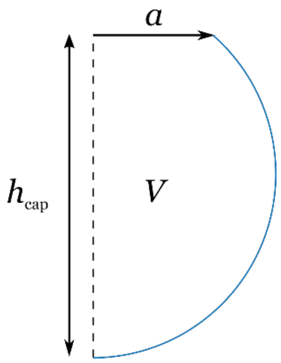

Supplementary Figure 11. Geometry of a spherical cap.

In this case:

$$\tilde{h}_{\text{snap-in}} = \tilde{h}_{\text{cap}} \quad (14)$$

where $\tilde{h}_{\text{cap}}$ can be solved geometrically

$$\tilde{h}_{\text{cap}} = k - \frac{1}{k}, \text{where } k = \left[\frac{3\tilde{V}}{\pi} + \sqrt{1 + \left(\frac{3\tilde{V}}{\pi}\right)^2}\right]^{1/3} \quad (15)$$

Supplementary Software 2 gives Matlab code to compute the snap-in force. Running this on a personal computer with a 2.6 GHz Intel i7 CPU takes approximately 4 s.

Supplementary Figure 12 compares the experimental data from micropillar measurements to simulated snap-in forces using Supplementary Equation 14. The model matches experimental data for the larger pillar radii, but does not match the experimental data for the smaller radii.

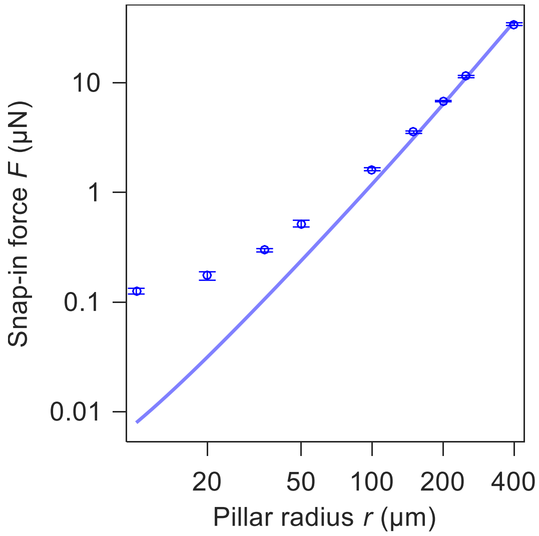

Supplementary Figure 12. Comparison between experimental snap-in force data and numerical results using Supplementary Equation 14.

The model described above assumes that the contact line pins to the top edge of the pillar cap. However, our micropillars have an undercut structure, and as we have previously reported [2], the contact line actually pins to the bottom edge of the pillar cap. In this case, the contact line wets the top and the sides of the pillar cap. Thus:

$$\tilde{h}_{\text{snap-in}} = \tilde{h}_{\text{cap}} + \tilde{h}_{\text{oxide}} \qquad (16)$$

where $\tilde{h}_{\text{cap}}$ is the height of the spherical cap and $\tilde{h}_{\text{oxide}}$ is the thickness of the oxide cap of the pillars, $h_{\text{oxide}} \approx 1.2$ µm for our pillars. Using Supplementary Equation 16, we get a much-improved match between the model and the data (Supplementary Figure 13). We conclude that the earlier discrepancy between the model and experimental data on the smallest pillars was an artefact of the undercut shape of the pillar tops; for flat samples, the model in Supplementary Fig. 12 is still expected to be more appropriate.

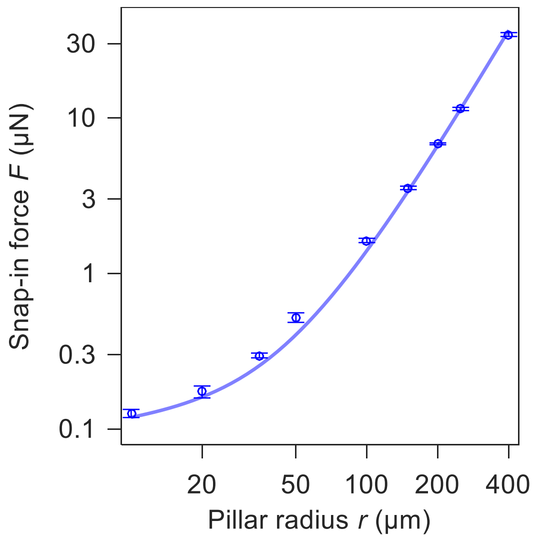

Supplementary Figure 13. Comparison between experimental snap-in force data and numerical results using (16). This is the same data as in Figure 2d.

Our experimental system could not observe small contact radii accurately, but the simulation model can be used to estimate the contact radius of the probed area for a given snap-in force, using the model in Supplementary Figure 12. The smallest snap-in force we have measured was 7.8 nN for the Hydrobead sample (Supplementary Table 2). Using the model in Supplementary Fig. 12, this corresponds to a contact radius of ~ 10 µm.

Furthermore, the model can also be used to predict how these values would change with the volume of the droplet. Supplementary Figure 14 shows the snap-in force as a function of droplet weight W for the case of $r = 100$ µm. The relative change $\left.\frac{dF}{dW}\frac{W}{F}\right|_{W=15\ \mu\text{N}} \approx -0.55$. During a typical measurement lasting for 20 s, the weight loss due to evaporation was observed to be around 1%, corresponding to an estimated increase of 0.55% in the snap-in force.

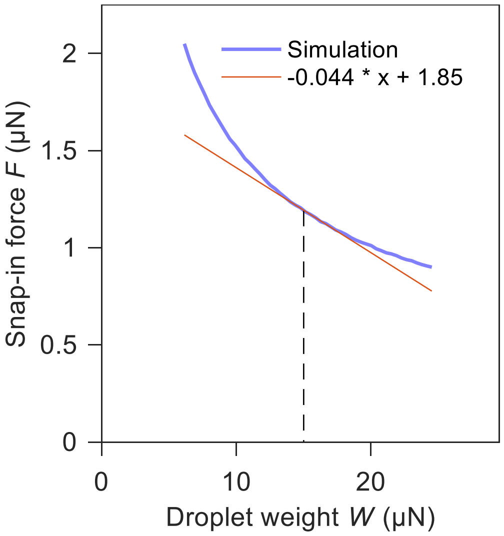

Supplementary Figure 14. Simulated relationship between droplet volume and snap-in force with $r = 100$ μm.

Pull-off forces on micropillars

We define the pull-off force as the maximum adhesive force with respect to $\tilde{h}$ i.e.

$$\tilde{F}_{\text{pull-off}}(\tilde{r}, \tilde{V}) = \max_{\tilde{h}} \tilde{F}(\tilde{h}, \tilde{r}, \tilde{V}) \tag{17}$$

A naïve implementation of Supplementary Equation 17 is numerically extremely inefficient, as within each step of the numerical optimization, we need to solve (12). It is much better to solve these problems simultaneously, as a constrained optimization problem:

$$\tilde{F}_{\text{pull-off}}(\tilde{r}, \tilde{V}) = \max_{\tilde{h}, x, \Delta\tilde{p}} \tilde{F}(\tilde{h}, x, \Delta\tilde{p}) \quad \text{s.t.} \quad \mathbf{m}(x, \Delta\tilde{p}) = \mathbf{0} \tag{18}$$

Supplementary Software 3 shows how to implement this in Matlab. The optimization starts from a distance near the snap-in distance, and we use the `force` function to find initial feasible values of x and $\Delta\tilde{p}$.

These values are in good agreement with the experiments on micropillars (Supplementary Figure 15).

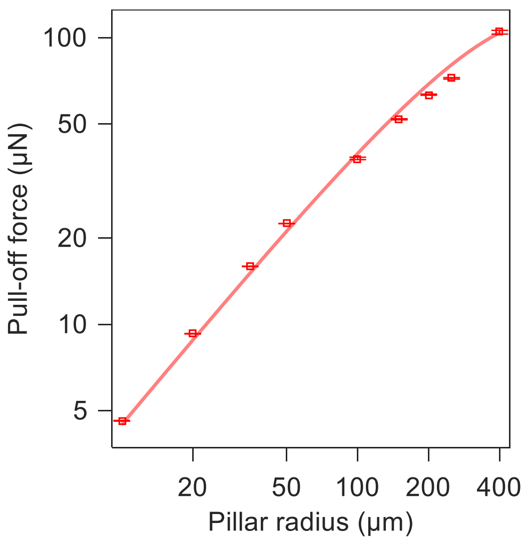

Supplementary Figure 15. Comparison between experimental pull-off force data and numerical results using (17). This is the same data as in Figure 2e.

Fixed angle problem

The fixed angle problem is

$$\begin{cases} \frac{u''}{(1+u'^2)^{3/2}} - \frac{1}{u\sqrt{1+u'^2}} = \Delta p/\gamma, \; z \in (0,h) \\ u'(0) = -\cot\theta \\ u(h) = a \\ \int_0^h \pi u^2 dz = V \end{cases} \qquad (19)$$

The only difference between the fixed angle problem and the fixed radius problem is that the contact angle, instead of contact radius, is defined at the substrate. The solution proceeds identically to the fixed radius problem: 1) non-dimensionalize; 2) find $\tilde{r}$ and $\Delta\tilde{p}$ using a shooting method; and 3) use (2) to compute the force. We omit repeating these steps for brevity.

This problem was studied in ref. [1] and our method recreates the numerical results from that paper (Supplementary Figure 16). There are some minor differences stemming from numerical inaccuracies, mostly at the limit when the meniscus becomes unstable. These inaccuracies do not affect the computed snap-in or pull-off forces.

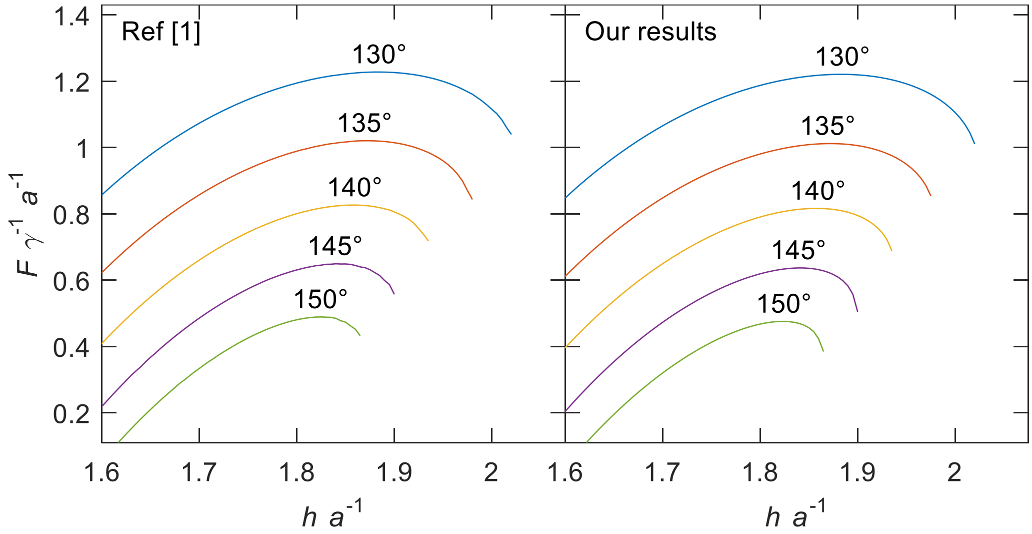

Supplementary Figure 16. Comparison between simulation results from [1] and results obtained using the method discussed here ($\tilde{V}$ = 5.44). Data from [1] courtesy of Hans-Jürgen Butt.

This model can be used to predict the relationship between the contact angle and snap-in / pull-off forces for ideal, smooth surfaces, using Supplementary Equation 14 for the snap-in distance. The model predicts that as the contact angle increases, both the snap-in force and the pull-off force decrease (Supplementary Figure 17).

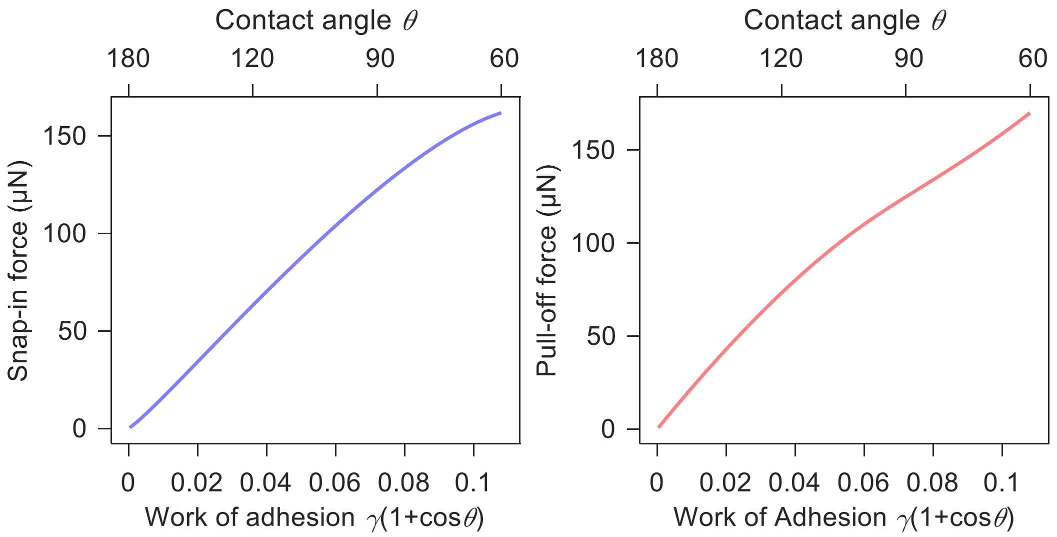

Supplementary Figure 17. Simulated relationship between snap-in and pull-off forces and work of adhesion / contact angle. When the contact angle increases, both forces decrease.

Supplementary References:

[1] Butt, H.-J. et al. Characterization of super liquid-repellent surfaces. *Curr. Opin. Colloid Interface Sci.* **19**, 343–354 (2014).

[2] Liimatainen, V., Sariola, V. & Zhou, Q. Controlling Liquid Spreading Using Microfabricated Undercut Edges. *Adv. Mater.* **25**, 2275–2278 (2013).
