# Supplementary material for: Mapping microscale wetting variations on biological and synthetic water-repellent surfaces
Source: Nat Commun. 2017 Nov 27;8:1798. doi: 10.1038/s41467-017-01510-7 (PMC5702616; doi:10.1038/s41467-017-01510-7)
Supplement: Supplementary file 3 — Description of Additional Supplementary Files [file 41467_2017_1510_MOESM3_ESM.pdf]

Description of Additional Supplementary Files

File Name: Supplementary Movie 1
Description: Scanning droplet adhesion microscopy: measurement procedure. The movie shows one measurement sequence of fully automated scanning procedure. The sample stage with butterfly wing starts fast moving up to approach the droplet (0.5 mm/s speed) followed by slow approach (5 µm/s speed) until the surface touches the droplet (snap-in moment). The sample stage continues moving up for a few seconds more (5 µm/s speed) after which retraction starts (10 µm/s speed) until the droplet separates from the butterfly wing (pull-off moment). Subsequently, sample stage moves down (0.5 mm/s speed), followed by droplet refill, grounding and moving to new measurement position.

File Name: Supplementary Movie 2
Description: Multiple wetting steps on a pillar surface. The movie shows side view of the experiment resulting in the force curve of Fig. 3b in the main text. Pinning (P1-P16) and depinning (DP1- DP8) events as the droplet advances/recedes on the pillar surface (5 µm radius, 70 µm spacing) are marked along the complete force curve shown at the bottom. The vertical blue line indicates current position on the curve, and the zoomed-in view on the top left, overlaid on the side view video, shows local details in the force. Playback of the movie is slowed down to 0.5X around the pinning and depinning events, and sped-up to 5X in between.

File Name: Supplementary Software 1
Description: Matlab code for computing the droplet adhesion force using the Simulink model of Supplementary Figure 10 and the shooting method.

File Name: Supplementary Software 2
Description: Matlab code for computing the snap-in force using the spherical cap approximation.

File Name: Supplementary Software 3
Description: Matlab code for computing the pull-off force using the “force” and “solveIVP” functions of Supplementary Software 1.
