# Supplementary material for: Mapping microscale wetting variations on biological and synthetic water-repellent surfaces
Source: Nat Commun. 2017 Nov 27;8:1798. doi: 10.1038/s41467-017-01510-7 (PMC5702616; doi:10.1038/s41467-017-01510-7)
Supplement: Supplementary file 6 — Supplementary Software 1 [file 41467_2017_1510_MOESM6_ESM.docx]

Matlab code for computing the force using the Simulink model and the shooting method.

% force.m file

function [Ft,zt,ut,du,pt] = force(rt,ht,Vt)

% set options for the fsolve algorithm

foptions = optimset('TolFun',1e-6,'TolX',1e-8,'Display','off','MaxIter',1000);

% fsolve finds the solution to the system of equations m = 0

% argm(1) = du/dz at z = 0, argm(2) = pressure

[argm,~,exitflag] = fsolve(@m,[0;1],foptions);

du = argm(1);

pt = argm(2);

% Force is the sum of laplace and capillary terms

Ft = 2 * pi * rt / sqrt(1+du^2) - pi * pt * rt^2;

% fsolve didn't converge, warn and return 0 instead of returning

% potentially erroneous values

if (exitflag <= 0)

warning('Did not converge, exitflag = %d',exitflag);

Ft = 0;

end

% Also return the shape of the drop that solves the BVP

[zt,ut] = solveIVP(rt,argm(1),argm(2),ht);

% Penalty is added only if the simulation ends before zt reaches ht

% i.e. if one of the conditional stops fires

function valm = m(arg)

[zt,ut,Vcurrent] = solveIVP(rt,arg(1),arg(2),ht);

penalty = 1+1000*(ht-zt(end));

valm = [Vt - Vcurrent;(1 - ut(end))*penalty];

end

end

% solveIVP.m file

function [zt,ut,V] = solveIVP(u0,du0,p,ht)

% Boilerplate to set u0, du0 and pressure in the Simulink model

assignin('base', 'u0', u0);

assignin('base', 'du0', du0);

assignin('base', 'p', p);

% Run the simulink model, 'time' is z in our case

simOut = sim('dropmodel',...

'StopTime',num2str(ht),...

'MaxStep',num2str(max(u0/400,1e-5)),...

'LimitDataPoints','off');

% Return the shape of the surface and volume of the drop

zt = simOut.get('tout');

yout = simOut.get('yout');

ut = yout(:,1);

V = yout(end,2);

% Close the system, this is needed for parallelization

close_system('dropmodel', 0);

end
