# Supplementary material for: Mapping microscale wetting variations on biological and synthetic water-repellent surfaces
Source: Nat Commun. 2017 Nov 27;8:1798. doi: 10.1038/s41467-017-01510-7 (PMC5702616; doi:10.1038/s41467-017-01510-7)
Supplement: Supplementary file 7 — Supplementary Software 2 [file 41467_2017_1510_MOESM7_ESM.docx]

Matlab code for computing the snap-in force using the spherical cap approximation.

a = 0.5e-3; % Radius of the probe disk [m]

gamma = 72e-3; % Surface tension [N/m]

W = 15e-6; % Weight of the droplet [N]

g = 9.81; % Gravitational acceleration [m/s^2]

rho = 1000; % Density of water [kg/m^3]

r = 400e-6; % Pillar radius [m]

% Non-dimensionalize volume and radius

V = W / (g*rho);

Vt = V / a^3;

rt = r / a;

% Use spherical cap approximation to compute the snap-in distance

k = nthroot(3*Vt/pi + sqrt(1 + (3*Vt/pi)^2),3);

ht_cap = k - 1 / k;

ht_snapin = ht_cap;

% Compute the snap-in force

F_snapin = gamma * a * force(rt,ht_snapin,Vt);
