# Supplementary material for: Mapping microscale wetting variations on biological and synthetic water-repellent surfaces
Source: Nat Commun. 2017 Nov 27;8:1798. doi: 10.1038/s41467-017-01510-7 (PMC5702616; doi:10.1038/s41467-017-01510-7)
Supplement: Supplementary file 8 — Supplementary Software 3 [file 41467_2017_1510_MOESM8_ESM.docx]

Matlab code for computing the pull-off force using the force and solveIVP functions.

function [Ft,zt,ut,du,pt,ht] = force_pulloff(rt,Vt)

k = nthroot(3*Vt/pi + sqrt(1 + (3*Vt/pi)^2),3);

ht_cap = k - 1 / k;

ht0 = ht_cap+0.06;

[~,~,~,du,pt] = force(rt,ht0,Vt);

x0 = [ht0;du;pt];

options = optimoptions(@fmincon,'Algorithm','sqp','Display','none', ...

'DiffMinChange',1e-4,'ConstraintTolerance',1e-3);

objfun = @(x) - 2 * pi * rt / sqrt(1+x(2)^2) + pi * x(3) * rt^2;

[x,~,exitflag] = fmincon(objfun,x0,[],[],[],[],[],[],@m,options);

% Force is the sum of laplace and capillary terms

Ft = -objfun(x);

ht = x(1);

du = x(2);

pt = x(3);

% fmincon didn't converge, warn and return 0 instead of returning

% potentially erroneous values

if (exitflag <= 0)

warning('Did not converge, exitflag = %d',exitflag);

Ft = 0;

end

[zt,ut] = solveIVP(rt,du,pt,ht);

function [c,ceq] = m(arg)

[~,utcurrent,Vcurrent] = solveIVP(rt,arg(2),arg(3),arg(1));

c = [];

ceq = [Vt - Vcurrent;1 - utcurrent(end)];

end

end
